# Nursing Education: Students’ Narratives of Moral Distress in Clinical Practice

**DOI:** 10.3390/nursrep11020028

**Published:** 2021-04-29

**Authors:** Marie Kvamme Mæland, Britt Sætre Tingvatn, Linda Rykkje, Sigrunn Drageset

**Affiliations:** 1 Faculty of Health Studies, VID Specialized University, Ulriksdal 10, 5009 Bergen, Norway; Britt.Tingvatn@vid.no (B.S.T.); linda.rykkje@vid.no (L.R.); 2 Department of Health and Caring Sciences, Western Norway University of Applied Sciences, Inndalsveien 28, 5063 Bergen, Norway; Sigrunn.Drageset@hvl.no

**Keywords:** moral distress, nursing students, clinical practice, education, narratives

## Abstract

Background: Research indicates that newly graduated nurses are often unprepared for meeting challenging situations in clinical practice. This phenomenon is referred to as a “reality shock”. This gap in preparedness may lead to moral distress. The aim of this article is to provide knowledge of moral distress in clinical nursing practice. Methods: Bachelor and further education nursing students were invited to write a story about challenging situations from their own clinical practice, resulting in 36 stories. Analysis was based on hermeneutical reading inspired by a narrative method; therefore, six stories were selected to represent the findings. Results: A finding across the stories is that the students knew the right thing to do but ended up doing nothing. Four themes were related to moral distress: (a) undermining of professional judgement, (b) disagreement concerning treatment and care, (c) undignified care by supervisors, and (d) colliding values and priorities of care. Conclusion: Nursing education should emphasize to a greater extent ethical competency and training for the challenging situations students will encounter in clinical practice.

## 1. Introduction

Research indicates that a gap exists between the academic preparation for a profession and the practical everyday challenges that the professionals face [1,2,3]. This is particularly challenging in the nursing profession, as students are often unprepared for meeting difficult situations that occur in practice, such as organizational issues, poor management, a lack of good role models, or demanding patient situations [4,5,6,7].

Stacey and Hardy [1] stated that the phenomenon of a “reality shock” appears to have been accepted as an inevitable aspect of professional socialization. Ajani and Moez [8] referred to this as the theory–practice gap in nursing, wherein nursing students may find themselves torn between the values they acquired at their learning institution and the reality of the clinical challenges [9,10,11]. The gap between what students have learned and the actions they take in practice may lead to “moral distress” [12]. Graduated nurses also experience moral distress [13,14,15]. In a systematic review by Sasso et al. [4], the authors highlighted that further research is needed to improve our understanding of the phenomenon of moral distress. Therefore, the focus in this study is upon students’ experiences of moral distress in clinical nursing practice.

McCarthy and Gastmans [12] argued that moral distress is an umbrella concept describing the psychological, emotional, and physiological suffering experienced when people act in ways that are inconsistent with deeply held ethical values, principles, or moral commitments. Jameton [16] described the concept of moral distress, defining it as the negative feelings that arise when one knows the morally correct response to a situation but cannot act, because of institutional or hierarchal constraints. After introducing the concept over 30 years ago, it has been proven applicable to many challenges in nursing practice [17].

Sasso et al. [4] suggested that nurses and nursing students are vulnerable to moral distress when faced with ethical dilemmas or decision making in clinical practice. Moral distress may manifest as anger, feelings of guilt and frustration, a desire to give up the profession, loss of self-esteem, depression, and anxiety. As a result, the nurse’s relationships with patients, families, and colleagues may be compromised. Helmers et al. [13] found that moral distress was a common experience in nursing practice, with both social and environmental stressors, such as lack of empathy or differences in opinion about care, prioritizing, or resource allocation. It seems that we cannot eliminate moral distress, thus we need to foster personal growth that enables nurses and students to better address and manage the situations of moral distress associated with practice [10,13]. Consequently, we need more knowledge about the types of difficult situations nurses encounter in their work.

The aim of this study is to provide knowledge of the experiences that can appear as examples of moral distress, based upon students’ written reflections about challenging experiences.

## 2. Materials and Methods

One class of bachelor students and two classes of further education nursing students at a Norwegian University College were asked to write about challenging situations from their own clinical practice. These written stories were analyzed using hermeneutic reading [18], inspired by the narrative method [19].

### 2.1. Data Collection and Participants

One of the teachers at the college provided written information about the project on a digital learning platform, in addition to oral information about the research project. The students were asked to do the following: “Write a story from clinical practice where you have experienced a situation that has upset you or challenged you”.

In the classroom setting, the students had 30 min to write down their story. They were also given the opportunity to finish the story at home. Each class of students submitted their stories anonymously through a digital learning platform. They were told to anonymize the stories and that no direct or indirect personal data should be described. In total, we received 36 stories: 19 stories from students in their third year of undergraduate nursing, seven stories from students specializing in oncology nursing, and 10 stories from students specializing in advanced gerontology.

The participants were Norwegian, except one person from Eastern Europe. Students undertaking the interdisciplinary course in gerontology were 16 women and one man, aged between 26 and 51 years. The oncology nursing students were 21 women, aged between 24 and 52 years. Students in their third year of undergraduate studies were 64 women and one man, aged between 21 and 38 years. As the submission of the written stories was anonymous, we cannot provide more details.

### 2.2. Research Ethics

Approval from the Centre for Research Data or Regional Ethical committee was not required as there were no direct or indirect personal data collected. The study was supported by institutional leaders. Submitting a story was considered as a formal consent to participate in the study. The students received oral and written information about the aim of the study, and they were guaranteed anonymity and were informed that they could withdraw from the study at any time. Furthermore, the students were asked not to include identifiable information about themselves or any persons involved in their stories. When presenting the selected stories, each was given a fictitious name.

### 2.3. Hermeneutic Reading Inspired by Narrative Method

Telling stories or narratives is an appropriate method for bringing up concrete subjective experiences, and has the potential to teach us more about the complex situations that are found in everyday life. In research based on narratives, individual stories can be highlighted to shed light on selected topics [19,20,21,22,23,24]. Teachers who participated in a “narrative research group” thoroughly read the 36 students’ stories. In the research group we discussed and categorized the kinds of stories that emerged. Many stories dealt with the topic of becoming a nurse, other stories dealt with moral distress in clinical practice. Therefore, the authors of this article decided to study more closely the phenomenon of moral distress.

Through hermeneutic reading [18], we looked for traces of moral distress across the 36 narratives, and after several rounds of reading each story in the dialectic hermeneutical movement between individual experiences and the material as a whole, an understanding emerged of how moral distress as a phenomenon can be recognized in practice. After reading the material several times it appeared that bachelor students and further education students shared similar stories dealing with moral distress in clinical practice. In the further analysis, we highlighted stories contributing with specific examples to help understand the phenomenon under study. Among these were moral distress stories dealing with the experiences of patients who were not offered the best treatment and care and stories related to the experience of poor guidance in clinical practice. What characterized the narratives as a whole was that the students knew the right action in the situation but ended up doing nothing.

As we read the stories again, we selected six narratives to illuminate the phenomenon of moral distress in clinical practice. We chose stories that both portrayed how students lack the power to act, and stories where students tried to act despite their limited opportunities to be heard. These narratives give insights into different facets of the challenges and dilemmas encountered in clinical practice, and we found that the stories represent central aspects of moral distress. The stories of the bachelor students and the nurses in further education were similar, therefore we chose to present the span and variety of the material, not the difference in educational level.

## 3. Results

There were four main themes related to moral distress in the narratives: (a) undermining of professional judgement, (b) disagreement concerning treatment and care, (c) undignified care by supervisors, and (d) colliding values and priorities of care.

### 3.1. Undermining of Professional Judgement

Not being heard by other professions, or being overlooked, are examples of difficult situations that students can encounter. Additionally, feeling that their professional judgment as nurses has been undermined can lead to moral distress. Several students wrote about challenges when collaborating with physicians, as portrayed in the narrative by Kari, a nurse working at the surgical department and specializing in advanced gerontology:

*Whilst working a night shift, I received a call from a woman who had recently been through surgery. She gave me a description of the problem she was having, and I recommended her to come immediately for a check-up. Approximately 20 min after speaking to the woman I received a call from an ambulance crew, sent to help a woman who had stopped her car and called the emergency services because she was bleeding heavily and felt dizzy. Once the ambulance arrived, I took her to the acute ward’s bathroom to assess the extent of the bleeding. Attempting to stop the bleeding, the woman had placed several towels and a bandage between her legs. All items were saturated with blood. The physicians were busy dealing with another patient and, after being made aware of the patient’s problems, instructed me to give the patient intravenous fluids.*

*The physicians were still busy when I informed them that the patient’s vital signs were fine, although I perceived the woman’s bleeding to be severe. Back in the patient’s room, her blood pressure was slightly lower and her pulse slightly higher. I knew that this meant that the blood pressure was adjusting to the loss of blood. I considered the situation to be urgent. The physician on duty assessed that it was still okay to have the patient in the surgical ward for observation. The patient repeated several times that she thought that she was going to die, but I replied that now she was in hospital and the bleeding would be stopped so she shouldn’t worry about dying. I was upset and scared, but I did what I could to hide my feelings from the patient. I called the physician on duty again and she wanted the patient back in the examination room. By then my patience was at an end, and I said, “The woman has been bleeding too much to move her yet again.” I thought the physician seemed uncertain and asked her to call the senior physician on duty, which she did. The senior physician decided that the woman should be sent for emergency surgery.*

*I felt like I had not been taken seriously. I felt that my patient had not been taken seriously. I felt like I had been left alone in a situation that I was not competent to handle and that no one had helped me. In retrospect, I have reflected on the situation and how I recall it. Maybe I did not communicate to the physicians how scared I was, or was too busy trying to appear calm and controlled towards the physicians? I will become clearer in my communication with physicians the next time I am in a similar situation.*

Kari felt unease related to her urgency and responsibility for the patient, yet she was unable to argue for her professional judgment because of too much respect towards the physicians’ opinions. Her story has similarities with the following stories by Sara and Monica, as they are concerned with disagreements and speaking up to physicians.

### 3.2. Disagreement Concerning Treatment and Care

Nurses are taught how to be the advocate of the patient; however, they often fall short in discussions about treatment and care with the physicians in charge. Several students experienced moral distress when witnessing that patients were not offered what they perceived as the best treatment and care, and they found it difficult to speak up about their concerns. Sara, a nurse specializing in cancer care, wrote:

*At a cancer ward, a young boy was very weak and was hardly able to speak. The patient had received cytostatic treatment for several years, and he knew that it was palliative treatment. What challenged me the most during a meeting with the patient and physician regarding future treatment choices was that the physician exclusively focused on the fact that if blood values improved, the patient could receive more cytostatic treatment. To me it seemed like the patient was prepared to talk about how soon he would be dying, and he stressed that he did not want further cytostatic treatment. I left this meeting with a bad feeling. Was there not anyone present who dared to talk with the boy about death? The patient died some days later. Was it just me as a student who was aware that we talked with a terminal patient?*

In the narrative written by Sara, we see her unease about the lack of communication about what mattered most to the patient’s current situation, that the patient did not want further cytostatic treatment and was prepared to die. Such conversations can cause moral distress in students who only sit by and are expected to be silent. Sara, as a continuous education student and experienced nurse, understood what the most important subject for the patient was, yet she was unable to speak up about her concerns.

In addition, the narrative of Monica, a third-year undergraduate nursing student, portrays a decision by the physician that impacted the quality of life of a very ill patient:

*A patient with cancer was considered by the doctor to have a poor prognosis. The patient became extremely angry when he was told that he should receive homecare nursing. The patient lived alone, had little family and friends, and Christmas was approaching. He told us that he was afraid to die alone and thought it was irresponsible of us to discharge him. I was left with a very bad feeling that we sent a terminally ill patient home alone for Christmas.*

Monica, who was only a spectator to this event, felt the emotional burden of the decision and the existential loneliness of a terminal patient who might end up dying at home alone during the holidays. Witnessing a lack of respect towards a helpless patient, without reacting in a way that could promote patient dignity, causes these kinds of incidents to remain as a strong memory that disturbs the student.

### 3.3. Undignified Care by Supervisors

Several students wrote about experiences of poor supervision. There is clearly a gap between what students learn about caring in a compassionate way and how they witness their supervisor or other experienced nurses practicing. The narrative of Elisabeth, a third-year undergraduate nursing student, portrays unprofessional nursing care:

*My first encounter with nursing practice was at a nursing home. My first task that morning was to care for a woman with dementia together with my supervisor. The way the supervisor walked into the patient’s room made me feel very uncomfortable. She opened the door without knocking. She turned on all the lights and said loudly: “Now you need to wake up!” It was awful how she woke up this helpless patient. I was thinking about what to do in this situation but ended up doing nothing. My supervisor performed tasks like she no longer thought about what she was doing, I thought she only acted on habit. This situation did something to me. I will never be like this! So terrible to be woken up this way.*

Elisabeth was very uncomfortable with the way the supervisor woke the patient, and that she seemed to act without compassion. In the situation the student thought about how to act, but she ended up doing nothing. We find that students may feel vulnerable being in such supervising situations, and that they, as the inferior party, may find it hard to speak up spontaneously. However, we may assert that the student learned from the experience, as she stated that she will never become such a nurse, only acting on routine. 

Similarly, Camilla, a nurse specializing in cancer care, wrote about a poor experience related to a supervision setting:

*A patient had given birth less than 24 h before. She had a lot of pain generally in her body. The supervisor asked me to insert a urinary catheter, she thought it could be a good training experience for me. I have done this a few times before but explained that I thought it was a bit worse when the patient had so much pain. I could not find the urethra and gave up very fast. In the end neither my supervisor nor myself could do it, we had to find another more qualified person. I thought that the patient would have suffered less if we from the start had contacted a nurse who was used to the procedure under these conditions.*

In the narrative Camilla understood that she could cause more pain to the patient, but although she suggested that the procedure would be difficult to perform, the supervisor was more interested in promoting the learning situation of the student than in preventing additional suffering and discomfort to the patient.

### 3.4. Colliding Values and Priorities of Care

Students learn to practice nursing based on the patient’s best interest. Therefore, experiencing managers making decisions and having priorities based on routines and resource allocation, based on availability, and not having the patient in focus, can be examples of “reality shock”. This reflective narrative was submitted by Martin, a student specializing in cancer care:

*A message was sent to the homecare team stating that the hospital wanted to arrange a meeting before the patient was discharged. This patient had not received homecare nursing before and therefore needed to be assured that the homecare nursing team had the necessary expertise and that someone from the homecare team would come to the hospital to meet the patient before she was discharged. The leader of homecare nursing in the municipality said that there was no need to visit the patient because they had such a well-established system. However, nurses in the homecare team were upset when they found out that the directive from the hospital was ignored by the leader. I found this episode very stressful because I know how important an interdisciplinary meeting at the hospital might be for the patient and her family in terms of feeling secure.*

The narrative above deals with organizational factors, in which leadership, management, and efficiency are prioritized in practice. However, the student did not try to speak up on behalf of the patient. These types of situations can be stressful to cope with and are examples of the gap between students’ academic preparation and the practical everyday challenges that professionals face when resources are scarce.

## 4. Discussion

The findings give insight into examples of how nursing students experience moral distress in clinical situations. The narratives demonstrate that the students felt discomfort and moral distress when they knew the best practice but found it difficult to speak up on behalf of the patient or to act to make a difference themselves [2,4,7,12]. The students describe a discrepancy between wishing to be the patient’s advocate and finding themselves in a position where they are afraid or unable to express their point of view. We encourage nursing education to raise awareness of such situations, where there is a gap between theory and practice, and to help students to improve their preparedness.

Kari’s story illustrates the importance of efficient and precise interdisciplinary communication for providing effective treatment and care to a critically ill patient. Collaboration across professions is a key component of creating environments that promote patient safety and quality nursing care [25,26]. Sara and Monica wrote about disagreement concerning treatment and care, and they portrayed how they ended up “doing nothing” despite knowing what the better course of action would be. One way of alleviating moral distress for the students might have been to communicate to the physicians what they had perceived as the wish of the patients. We acknowledge that novices might not understand the reasoning behind how senior nurses or doctors act, and students are not always the ones who know the right thing to do. Nevertheless, students will experience conflicts between what they learn or perceive as good care and what is provided. If students are not prepared for encountering these kinds of situations, they may feel moral distress [2,13].

Elisabeth’s and Camilla’s stories are examples of supervisors providing poor care to patients. Elisabeth was a spectator when a resident with dementia was poorly treated. Sara tried to explain that she did not feel competent to insert a urinary catheter since the patient was in pain, but finally the supervisor also had to give in and find a more experienced nurse to perform the procedure. Selecting the right person to be a student supervisor in clinical practice is crucial [27]. To serve as good role models, supervisors need to have sufficient knowledge and training to guide students. Offering regular guidance courses for supervisors, organized by the college, would be an important contribution to decreasing the gap between theory and practice. Moreover, a completed guidance course should be mandatory to be approved as a clinical supervisor. According to Driscoll et al. [28] and Wilson [29], supervisors must have high levels of moral autonomy, the responsibility to take the morally correct action for a patient, and moral judgment, which involves the supervisor’s ability to consider both sides of a moral situation and determine the best course of action.

Martin’s story illustrates that colliding values and priorities of care interventions often come into conflict with the interpersonal values of compassion, empathy, and making time for the patient [9,11]. Indeed, dilemmas in healthcare services are increasingly related to tensions between financial constraints and management strategy on the one hand, and individual ethical ideals on the other [30]. According to Huffman and Rittenmeyer [2], healthcare systems with fewer financial resources, weak policies, and poor staffing contribute to the development of moral distress in nurses. Wilson [29] argued that we need to highlight the impact that organizational constraints have on moral distress, causing nurses with less organizational support and resources to be at higher risk of experiencing moral distress.

The narratives in this study support other research finding that bachelor and further education nursing students appear vulnerable to moral distress when faced with ethical dilemmas and decision making in clinical practice [4]. A finding across the stories is that the students knew the right thing to do but found it difficult to act. It is important to question whether nurses have the moral judgment and wisdom to really know the right thing to do [17], an important issue we believe should be addressed in nursing education. In addition, when encountering challenging situations, both nursing education and practice need to promote interprofessional collaboration and ethical competence to foster “moral courage” [31]. We suggest that methods other than just classroom teaching may empower students to be better prepared for challenging situations. For example, simulation of ethical dilemmas is one method for practicing a patient situation that is as real as possible, without exposing a patient to risk. Simulation facilitates interactions between participants working towards specific learning goals in a simulated real-life setting. This method is also suitable for exercising interdisciplinary collaboration and communication in healthcare [32,33].

Our findings reveal that students may blame themselves for not daring to be clear enough with other health professionals, especially physicians, when they see that the patient is not adequately safeguarded [34]. There may be several reasons for this. Hospitals often have a hierarchical structure, and the students are near the bottom because of their status. In addition, the students’ roles may be demanding, as they are constantly evaluated in clinical practice, which may cause uncertainty about doing a good enough job [7,35]. Thus, we find that being self-judgmental may influence their evaluation in a negative way. This leads to moral distress because the student has a clear understanding of the situation but is nonetheless reluctant to intervene or speak up for the patient.

The healthcare system is often characterized by efficiency. Consequently, the patient may easily be treated as a medical object because healthcare professionals may sometimes not have time for his or her personal concerns. Furthermore, in Norway, increased outpatient treatment and reduced hospital stays cause health professionals to have less time and opportunity to provide care [36]. This makes it even more important to be present in brief encounters with patients and to be aware of their reactions and experiences [37]. The challenge for educators is to prepare students for the reality of today’s health care services and for the limited possibility of delivering the best possible care at the standards students learn at school.

At our college, bachelor nursing students and students in further education programs are required to write narrative reflection notes from their clinical practice [38]. These stories are often moral distress stories, and we find it important that students develop the skill of critically reflecting on such clinical ethical challenges [39]. One educational method we find promising, which may counterbalance moral distress [40,41], is the so-called “reflection group”, where the students are divided into smaller groups to share demanding clinical experiences with each other and the teacher. This method can be suitable for ethical reflection and for preparing students for demanding clinical situations. However, a pitfall one must be aware of is not recognizing institutional and professional failures, and rather putting the focus on individual nurses and nursing students to adapt themselves to what may be a destructive environment. According to Monteverde [42], it is important to enable students to understand and master the moral stressors they experience in practice or learn about from others. Moreover, when identifying what is morally wrong it is also important to enable students to change their course of action despite adverse consequences at a personal and systemic levels [42]. Our recommendation is to strengthen students’ ability to critically reflect on their experiences, which might also help in closing the practice–theory gap and increase their preparedness for clinical practice.

### Strengths and Limitations

The students’ narratives gave insight into their current clinical experiences and reflections. In line with Frank [19] and Riessman [24], using narratives can be characterized as qualitative analysis, and each story is perceived as a fundamental source to examine human experiences, actions, and understandings. According to Frank [19,22], stories accompany people through their lives, and we find that telling stories can help the students become aware of what counts as right or wrong, and teach them who they are and who they want to be.

Furthermore, people tell different stories in different settings because the setting influences the story [6,22]. Therefore, it is essential to bear in mind that each student’s narrative is situational and aimed at a specific audience. Our students were familiar with the requirements regarding the writing of reflective narratives on ethical challenges in clinical practice. Thus, they were not unfamiliar with the task they were given in this study.

The authors of this article are experienced nurses and teachers, responsible for guiding nursing bachelor and further education students. We see this as an advantage, as we are well acquainted with different patients and environments that students may encounter in clinical practice. On the other hand, this could also be a disadvantage, as the authors might have “blind spots” when analyzing the narratives. Therefore, it was important for the authors to read the narratives independently and to discuss the findings in several meetings before agreeing upon the final themes.

With qualitative methods, broad inferences cannot be drawn. The sample consists of students at a Norwegian college, which also limits the transferability of the findings. However, the narratives in this article provide a rich, in-depth understanding of nursing students’ experiences of moral distress.

## 5. Conclusions

The students’ narratives add to the existing knowledge about moral distress in clinical practice, and we challenge nursing education and clinical practice to address students’ experiences of moral distress. Nursing education should emphasize to a greater extent ethical competency and training for the challenging situations students will encounter in clinical practice. This may mitigate the negative consequences of moral distress. Furthermore, good role models in clinical practice and organized guidance and support will increase self-reliance and could minimize the gap between academic preparation and challenges in nursing practice.

An improved understanding of students’ experiences of moral distress is a first step towards empowering them when facing challenging situations. Through enhanced teaching methods, we suggest how nursing students can become better prepared for the reality they encounter in clinical practice. Furthermore, closer collaboration between teachers at colleges and interdisciplinary teams in clinical practice may help to reduce the gap between theory and practice.
